# Chemotherapy induced nausea and vomiting may cause anxiety and depression in the family caregivers of patients with cancer

**DOI:** 10.3389/fpsyt.2023.1221262

**Published:** 2023-09-20

**Authors:** Xiaozhen Luo, Lili Yang, Jing Chen, Jing Zhang, Quanqing Zhao, Jiang Zhu

**Affiliations:** ^1^Department of Medical Oncology, Shangjin Nanfu Hospital, West China Hospital, Chengdu, China; ^2^Department of Thoracic Oncology, West China Hospital, Sichuan University, Chengdu, China

**Keywords:** highly emetogenic chemotherapy (HEC), chemotherapy induced nausea and vomiting (CINV), family caregivers, anxiety, depression

## Abstract

**Objective:**

To investigate the impact of chemotherapy induced nausea and vomiting (CINV) on the anxiety and depression of the primary family caregivers of patients with cancer.

**Methods:**

This study screened family caregivers of patients with cancer undergoing highly emetogenic chemotherapy (HEC) containing a 3-day cisplatin regime. Caregivers who did not experience anxiety or depression at baseline screening were enrolled in this study. Based on the patients’ CINV status during chemotherapy, their family caregivers were divided into two groups: patients who experienced CINV (CINV group) and patients who did not experience CINV (No-CINV group). All enrolled family caregivers completed the Hospital Anxiety and Depression Scale (HADS) questionnaire on the fourth and 8 days of chemotherapy.

**Results:**

A total of 256 family caregivers were screened for this study, of which 195 caregivers without anxiety or depression at baseline were included. A total of 150 (76.9%) patients undergoing chemotherapy experienced acute CINV; 63 (42%) of their family caregivers experienced anxiety, and 65 (43.3%) developed depression. This was significantly higher than the experiences of the No-CINV group (2.2%, *P* < 0.001; 0%, *P* < 0.001, respectively). Among the patients undergoing chemotherapy, 86 (44.1%) experienced delayed CINV. The incidence of anxiety and depression in the family caregivers of patients with delayed CINV were 27.9 and 36%, respectively, both of which were significantly higher than that in the family caregivers of the No-CINV group (0.9%, *P* < 0.001; and 0.9%, *P* < 0.001, respectively).

**Conclusion:**

Acute and delayed CINV occurring in patients during chemotherapy may induce anxiety and depression in their family caregivers.

## Introduction

Chemotherapy remains an importance in the treatment of cancer, but chemotherapy induced nausea and vomiting (CINV) is one of the most common and most feared toxic reactions for patients receiving chemotherapy (1). In addition to severely affecting patients’ quality of life, CINV can cause metabolic disorders, malnutrition, and weight loss in cancer patients, significantly increasing their physical and psychological burdens and leading to a decrease in treatment compliance (2, 3). Previous randomized clinical trials (RCT) have shown that, through effective prevention procedures, the incidence of acute CINV is about 10–20% and that of delayed CINV is about 25–30% (4). However, it is reported that, in a real-world setting, the incidence of acute CINV is about 40–55% and that of delayed CINV can be as high as 50–60% (5, 6). CINV has a significant impact on patients with cancer in clinical practice (2), but there is currently no relevant research data to explore the existence of any adverse effects on their family caregivers.

Family members are the main caregivers for patients with cancer, with spouses, children, and relatives being the most common family caregivers. The huge shock resulting from the diagnosis of cancer of a family member can often exacerbate the physical and psychological burdens of the family caregivers, which can cause further anxiety and depression (7). Interestingly, the mental state of family caregivers can in turn affect the patient’s mental state (8). However, so far, there is currently a lack of research on the impact of CINV on the family caregivers of cancer patients. This study aims to preliminarily explore whether the occurrence of CINV in patients with cancer during chemotherapy influences the psychological status of their primary family caregivers.

## Materials and methods

### Research subjects

This is a prospective, non-interventionist, real-world study. The family caregivers of cancer patients who were hospitalized in our oncology center and received a 3-day cisplatin-containing highly emetic chemotherapy (HEC) were screened. Based on the patient’s CINV status during chemotherapy, their family caregivers were divided into two groups: those who experienced CINV (CINV group) and those who did not (No-CINV group). All eligible family caregivers completed the Hospital Anxiety and Depression Scale (HADS) questionnaire on fourth and 8 days of chemotherapy.

The inclusion criteria were as follows: (1) The accompanied patient was diagnosed with cancer; (2) The accompanied patient was scheduled to receive a 3-day cisplatin-containing HEC during the current hospitalization; (3) The family caregiver would not change during the patient’s current hospitalization; (4) The family caregiver had normal intelligence and understanding, and could complete the questionnaire independently; (5) The family caregiver had voluntarily participated in this research; (6) HADS score was 0∼7 in each subscales. The exclusion criteria were as follows: (1) The caregiver was a professional; (2) There were known conflicts between the family caregiver and the patient; (3) The family caregiver had mental and cognitive abnormalities; and (4) The family caregiver had a serious illness. This study was proved by the Ethic Committee of Shangjin Nanfu Hospital West China Hospital, and was registered in the world health organization (WHO) international clinical trials registered organization registered platform (https://www.chictr.org.cn; ChiCTR2300069307).

### Research tools

General demographic questionnaire: This included basic information such as the caregiver’s name, gender, age, whether the patient was an immediate family member, whether the patient had social security, whether the caregiver was a local, whether the patient was new to treatment, and the patient’s PS score.

MASCC Antiemetic evaluation tool table (MAT): This tool was used to assess the occurrence of acute CINV (completed on day 4 of chemotherapy) and delayed CINV (completed on day 8 of chemotherapy) in patients (9). Patients who did not experience vomiting and/or significant nausea and had no record of rescue antiemetic were classified as “no CINV”; their family caregivers were assigned to No-CINV group. If the patients had experienced vomiting and/or significant nausea, or given relief from rescue antiemetic, were classified as “CINV”; their family caregivers were assigned to CINV group.

Hospital Anxiety and Depression Scale (HADS): This scale was used to assess the anxiety and depression status of family caregivers on the fourth and 8 days of chemotherapy. The HADS had consisted of 14 items and were divided into two subscales for anxiety and depression, with each containing seven items. Each item was rated on a scale of 0 to 3, with the total score ranging from 0 to 21. A score of > 7 was used as a criterion for the occurrence of anxiety/depression, with a score of 8–21 indicating an occurrence of anxiety/depression and that of 0–7 indicating the absence of anxiety/depression, with 8–10 being mild anxiety and/or depression, 11–14 being moderate anxiety and/or depression, and 15–21 being severe anxiety and/or depression (10).

### Statistical methods

The data was analyzed using the SPSS 20.0 statistical software package (IBM Corp., Armonk, NY, USA). The count data was expressed as the number of cases (in percentage), and the χ^2^ test was used for comparison between groups. Normally distributed measurement data was represented by the mean ± standard deviation (x ± s), and two independent sample *t*-tests were used for comparison between groups, with *P* < 0.05 being considered statistically significant.

## Results

### Baseline characteristics

**FIGURE 1:**
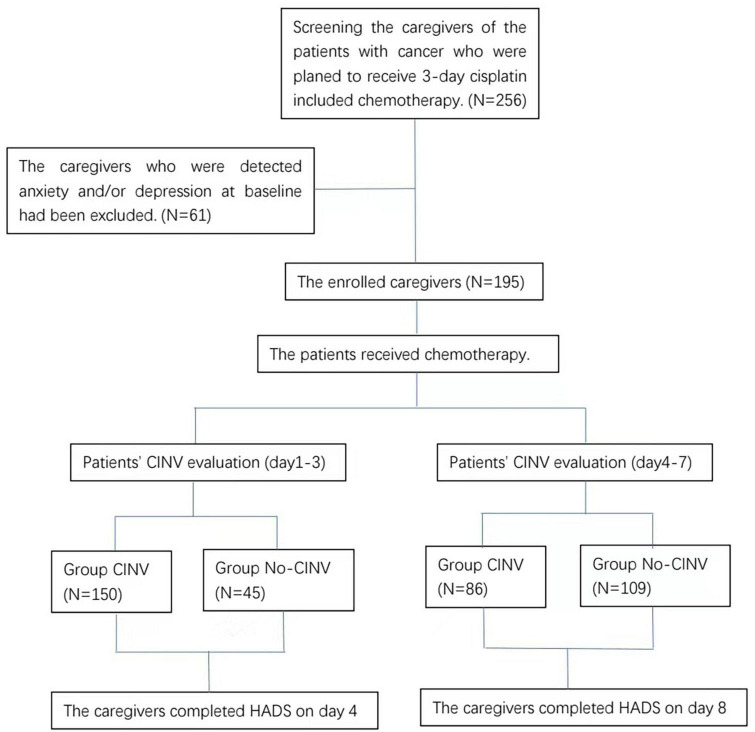
Flow chat of the study.

A total of 256 family caregivers were screened for this study, of which 61 were excluded due to existing anxiety and depression assessed during baseline evaluation. The remaining 195 family caregivers who had not yet experienced anxiety and/or depression were included in the study (Figure 1). There were no statistical differences in the general demographic characteristics of the two groups (Table 1).

**TABLE 1:** The characteristics of the caregivers.

Characteristics		Day 4			Day8	
	**Group**	**Group**	***P-*value**	**Group**	**Group**	***P-*value**
	CINV	No-CI NV		CINV	No-CI NV	
Gender			0.602			0.242
Male	56	19		38	39	
Female	94	26		48	70	
Median age	51	50	–	51	50	–
Immediate family member			0.591			0.386
Yes	145	45		85	105	
No	5	0		1	4	
Education level			0.847			0.876
Primary school and below	53	18		34	41	
Junior/High School	69	19		42	57	
College degree or above	28	8		10	11	
Health insurance for the patient			0.799			0.519
Yes	132	39		77	94	
No	18	6		9	15	
Native residents			1.000			0.544
Yes	92	28		54	74	
No	58	17		32	35	
Chemotherapy naive			0.810			0.442
Yes	21	7		17	16	
No	129	38		69	93	
Patients’ performance status score (ECOG)			0.222			0.200
0	120	32		66	92	
1	30	13		20	17	
Pathologic diagnosis			0.225			0.698
Lung cancer	41	20		28	34	
Cervical carcinoma	32	6		21	25	
Ovarian cancer	37	9		14	26	
Esophageal cancer	33	7		18	17	
Other cancer	7	3		5	7	
TNM Stage			1.000			0.484
I	7	2		6	3	
II	29	9		19	21	
III	67	20		38	55	
IV	47	14		23	30	

### Analysis of anxiety/depression in family caregivers affected by acute CINV in the two groups

**FIGURE 2:**
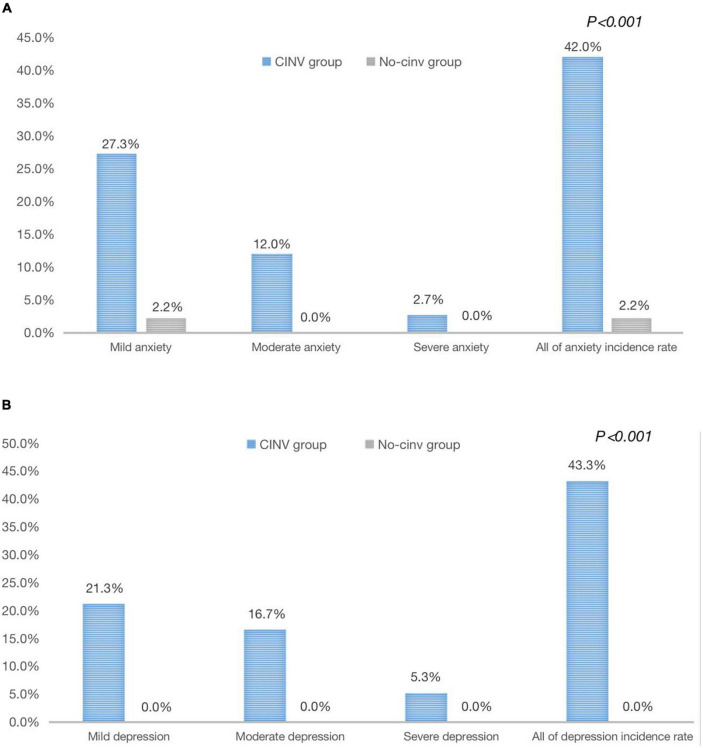
Analysis of anxiety/depression in family caregivers affected by acute CINV in the two groups. **(A)** Analysis of anxiety in family caregivers affected by acute CINV in the two groups. **(B)** Analysis of depression in family caregivers affected by acute CINV in the two groups.

In the study, 150 patients (76.9%) experienced acute CINV (day 1∼day 3); among the caregivers of these patients, their average HADS scale was 6.65 ± 3.47 in anxiety and 7.75 ± 3.85 in depression. 63 (42%) were found to experience anxiety according to the assessment on the fourth day of chemotherapy, and 65 (43.3%) developed depression, which were significantly higher than those in the No-CINV group (2.2%, *P* < 0.001; 0%, *P* < 0.001, respectively), and their average HADS scale was 1.80 ± 1.79 in anxiety and 2.40 ± 1.68 in depression (Figure 2).

### Analysis of anxiety/depression in family caregivers induced by delayed CINV in both groups

**FIGURE 3:**
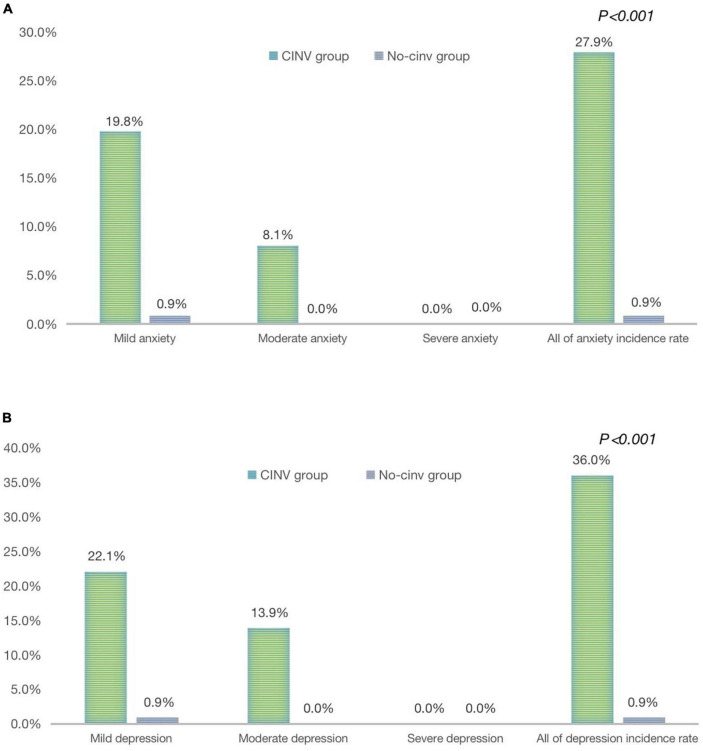
Analysis of anxiety/depression in family caregivers include by delayed CINV in the both groups. **(A)** Analysis of anxiety in family caregivers induced by delayed CINV in both groups. **(B)** Analysis of depression in family caregivers induced by delayed CINV in both groups.

In total, 86 patients (44.1%) experienced delayed CINV (day 4∼day 7) in this study, their average HADS scale was 6.57 ± 3.37 in anxiety and 5.67 ± 2.95 in depression. In the CINV group, 24 family caregivers (27.9%) were found to have anxiety, and 31 (36%) had depression on the 8 day of chemotherapy, which were significantly higher than those in the No-CINV group (0.9%, *P* < 0.001; 0.9%, *P* < 0.001, respectively), and their average HADS scale was 1.15 ± 1.37 in anxiety and 2.01 ± 1.54 in depression (Figure 3).

## Discussion

There is currently no clear international consensus on the definition of family caregivers, also known as informal caregivers. Some difference in expression about the family caregivers exists in different organizations (11, 12). In summary, a family caregiver can be defined as a person who provides continuous care and assistance to a family member in need of external support due to poor physical, cognitive, or mental health, without receiving remuneration. This includes relatives, partners, and other individuals providing care to the person in need.

It is generally believed that family caregivers have a responsibility to care for the patients with cancer (13). Caring for patients with cancer brings changes to the caregiver’s existing work, life, and the entire family. Many caregivers shift their focus to caring for and accompanying patients through multiple cycles of anti-cancer treatment, and are forced to adjust their existing lives and work to accommodate these changes (14). However, this also challenges the original family and social roles of the caregiver, adding new responsibilities and burdens. Long-term care for patients also causes family caregivers to suffer from problems such as sleep disorders, physical exhaustion, and psychophysiological issues (15). This process can be very challenging for family caregivers (16).

China has predicted that the overall 5-year cancer survival rate will be no less than 46.6% by 2030 (17). Thus, the number of cancer survivors and their family caregivers will increase rapidly, which is a cause for concern. Currently, many cancer patients have problems such as advanced age, long duration of illness, and multiple complications, which prevent them from managing their illness independently (18). Family members play a key role in the caregiving process while bearing a huge burden that seriously affects their quality of life and physical and mental health (19). Gupta found that 43% of caregivers would need to take a break from long-term caregiving tasks and shorten their caregiving time to recover physically and mentally (20). Only when the caregivers are physically and mentally healthy can the quality of care be improved and the recovery of patients be facilitated. Therefore, it is crucial to pay attention to the family caregivers of patients with cancer.

Yang et al. (21) found that 29.83% of family caregivers might have had anxiety, and 28.73% of family caregivers might have had depression 24 h before the start of the patient’s first chemotherapy treatment, with a higher incidence and level of anxiety and depression in family caregivers of patients with cancer undergoing chemotherapy. Studies based on the trajectory of the illness of cancer patients have found that 40–55% of cancer family caregivers were significantly depressed and 30–58% of family caregivers were depressed, with associated risk factors including caregiving time, financial stress, age, and social support (22–24). Quality of life of patients with cancer greatly affects that of the caregivers (25), and the quality of life of caregivers of patients with cancer during chemotherapy is worse than that of caregivers of general patients (26).

The side effects of chemotherapy may be an important reason for affecting the mental health of family caregivers (26). Despite great advances made in the prevention of emesis, CINV remains one of the most common and serious toxic side effects of chemotherapy (2). This study showed that the incidence of acute CINV was 76.9%, and the incidence of delayed CINV was 44.1%, which is higher than previous RCT data. The possible reasons for the analysis are as follows: (1) This study is a non-interventional real-world study that does not provide regulations for CINV prevention, so there is a lack of prevention in clinical practice; (2) The chemotherapy included in the study was a 3-day regimen containing cisplatin; and (3) Another possible reason was that the treatment completion and cooperation level of patients in the real world were not as good as those in clinical trials. In this study, acute CINV was defined as the first to third day of chemotherapy, and delayed CINV was defined as the fourth to seventh day of chemotherapy, which is different from the RCT of single day chemotherapy. CINV significantly reduces quality of life of patients, and in severe cases, it can cause metabolic disorders, organ function impairment, reduced nutrition and physical fitness, and discontinuation of anti-tumor treatment (2, 6). The adverse effects of CINV on patients with cancer are clear. However, does CINV increase the burden on family caregivers and lead to a negative impact on their psychological state? Currently, research data is scarce. The results of this study showed that the prevalence of anxiety was 42% and that of depression was 43.3% in the family caregivers if acute CINV occurred in patients with cancer undergoing chemotherapy. If delayed CINV occurred in chemotherapy patients, anxiety was experienced by 27.9% of and depression by 36% of family caregivers, both of which were significantly higher than that experienced by family caregivers of patients who did not have CINV during chemotherapy. The above results suggest that the occurrence of CINV in patients with cancer undergoing chemotherapy may induce anxiety/depression in their family caregivers, thus increasing their burden and negatively impacting their mental health. This phenomenon deserves further attention and in-depth analysis in future studies.

More than 60% of CINV can be effectively prevented if standardized prophylactic antiemetic treatments were given to patients according to the relevant guidelines (27). In addition, optimized anti-emetic protocols, such as Chinese herbal medicine, can be adopted to enhance the preventive efficacy (28, 29). Based on the data of this study, adopting practical and effective prevention plans will greatly reduce the incidence of CINV in chemotherapy patients, which may reduce the risk of anxiety/depression among family caregivers.

In summary, the results of this study showed that CINV might induce anxiety and depression in family caregivers of patients with cancer, which derives more attention to enhance their quality of life. This was a single-center study with limited sample sources and the possibility of selection bias. Therefore, further in-depth research is needed to overcome these limitations.

## Conclusion

The occurrence of CINV in patients with cancer undergoing chemotherapy may induce anxiety/depression in their family caregivers, and this phenomenon deserves further investigation.

## Data availability statement

The raw data supporting the conclusions of this article will be made available by the authors, without undue reservation.

## Ethics statement

The studies involving human participants were reviewed and approved by the Ethics Committee of Shangjin Nanfu Hospital, West China Hospital of Sichuan University. The patients/participants provided their written informed consent to participate in this study. Written informed consent was obtained from the individual(s) for the publication of any potentially identifiable images or data included in this article.

## Author contributions

JZu and XL designed and conducted this study. LY, JZa, JC, and QZ screened the subjects, completed the questionnaires, and processed the follow up. XL and LY performed the data analysis. LY, XL, and JZu drafted the manuscript. All authors contributed to the article and approved the submitted version.

## References

[1] Razvi Y Chan S McFarlane T McKenzie E Zaki P De Angelis C ASCO, NCCN, M/ESMO: a comparison of antiemetic guidelines for the treatment of chemotherapy-induced nausea and vomiting in adult patients. *Support Care Cancer.* (2019) 27:87–95. 10.1007/s00520-018-4464-y 30284039

[2] Aapro M. CINV: still troubling patients after all these years. *Support Care Cancer.* (2018) 26:5–9. 10.1007/s00520-018-4131-3 29556808 PMC5876280

[3] Seyedfatemi N Ghezeljeh TN Bolhari J Rezaei M. Effects of family-based dignity intervention and expressive writing on anticipatory grief of family caregivers of patients with cancer: a study protocol for a four-arm randomized controlled trial and a qualitative process evaluation. *Trials.* (2021) 22:751. 10.1186/s13063-021-05718-3 34711262 PMC8552199

[4] Aapro M Carides A Rapoport BL Schmoll HJ Zhang L Warr D. Aprepitant and fosaprepitant: a 10-year review of efficacy and safety. *Oncologist.* (2015) 20:450–8. 10.1634/theoncologist.2014-0229 25795636 PMC4391760

[5] Liau CT Chu NM Liu HE Deuson R Lien J Chen JS. Incidence of chemotherapy-induced nausea and vomiting in Taiwan: physicians’ and nurses’ estimation vs. patients’ reported outcomes. *Support Care Cancer.* (2005) 13:277–86. 15770489 10.1007/s00520-005-0788-5

[6] Sun Y Zheng Y Yang X Xie K Du C He L Incidence of chemotherapy-induced nausea and vomiting among cancer patients receiving moderately to highly emetogenic chemotherapy in cancer centers in Sichuan, China. *J Cancer Res Clin Oncol.* (2021) 147:2701–8. 10.1007/s00432-021-03554-1 33586045 PMC11802099

[7] Unsar S Erol O Ozdemir O. Caregiving burden, depression, and anxiety in family caregivers of patients with cancer. *Eur J Oncol Nurs.* (2021) 50:101882. 10.1016/j.ejon.2020.101882 33421929

[8] Del-Pino-Casado R Priego-Cubero E López-Martínez C Orgeta V. Subjective caregiver burden and anxiety in informal caregivers: a systematic review and meta-analysis. *PLoS One.* (2021) 16:e0247143. 10.1371/journal.pone.0247143 33647035 PMC7920375

[9] Matsuda Y Okita K Furuhata T Kutomi G Yamashita K Sato Y Evaluation of the validity of chemotherapy-induced nausea and vomiting assessment in outpatients using the Japanese version of the MASCC antiemesis tool. *Support Care Cancer.* (2015) 23:3331–9. 10.1007/s00520-015-2780-z 26003425

[10] Annunziata MA Muzzatti B Bidoli E Flaiban C Bomben F Piccinin M Hospital Anxiety and Depression Scale (HADS) accuracy in cancer patients. *Support Care Cancer.* (2020) 28:3921–6. 10.1007/s00520-019-05244-8 31858249

[11] Family Caregiver Alliance. *Caregiver Statistics: Health, Technology and Caregiving Resources.* San Francisco, CA: Family Caregiver Alliance (2023).

[12] The National Alliance for Caregiving. *Care for the Family Care-Giver: A Place to Start.* Washington, DC: The National Alliance for Caregiving (2023).

[13] Wang S Jiarong L Changying L Tianying Y Xiaoxuan L Mingxia C. Analysis of research hotspots and frontiers on psychology of cancer family caregivers. *J Clin Med Pract*. (2022), 26:105–10, 114. 10.7619/jcmp.20221416

[14] Chua GP Pang GSY Yee ACP Neo PSH Zhou S Lim C Supporting the patients with advanced cancer and their family caregivers: what are their palliative care needs? *BMC Cancer.* (2020) 20:768. 10.1186/s12885-020-07239-9 32799834 PMC7429720

[15] Jixing Y Shenpan L Junying H Xiang L. Status quo of anxiety and its influencing factors among family caregivers accompanying 226 patients with cancer. *Pract Prevent Med*. (2022) 29:410–3. 10.3969/j.issn.1006-3110-2022004007

[16] Kajiwara K Kako J Noto H Oosono Y Kobayashi M. Zarit Burden Interview in the palliative care setting. *Support Care Cancer.* (2020) 28:3479. 10.1007/s00520-020-05471-4 32318869

[17] Liu Z Li Z Zhang Y Zhou T Zhang J You W Interpretation of the Global Cancer Statistical Report in 2020. *Online Magaz Compr Cancer Treat.* (2021) 7:1–14.

[18] Parmelee Streck B LoBiondo-Wood G. A systematic review of dyadic studies examining depression in couples facing breast cancer. *J Psychosoc Oncol.* (2020) 38:463–80. 10.1080/07347332.2020.1734894 32202229

[19] Wan JBP Rq L. Effects of positive stress reduction therapy on negative emotions and caregiving burden of accompanying family members of depressed patients. *Chin Natl Health Med.* (2020) 32:55–7.

[20] Gupta K Walton R Kataria SP. Chemotherapy-induced nausea and vomiting: pathogenesis, recommendations, and new trends. *Cancer Treat Res Commun.* (2021) 26:100278. 10.1016/j.ctarc.2020.100278 33360668

[21] Yang M Lan B Sun XY Ma F Cai JQ Bh X. Analysis of the psychological status of 181 family caregivers of postoperative adjuvant chemotherapy patients with breast cancer. *Chin J Clin Phys.* (2020) 48:1059–62.

[22] Jing C Shuang Y. Investigation on mood of caregivers of patients with hematological malignancies and its influential factors. *J Nurses Train*. (2019) 34:303–8.

[23] Luo J Zhou Y Liu H Hu J. Factors related to the burden of family caregivers of elderly patients with spinal Tumours in Northwest China. *BMC Neurol.* (2020) 20:69. 10.1186/s12883-020-01652-0 32111172 PMC7047359

[24] Segrin C Badger TA Sikorskii A Pasvogel A Weihs K Lopez AM Longitudinal dyadic interdependence in psychological distress among Latinas with breast cancer and their caregivers. *Support Care Cancer.* (2020) 28:2735–43. 31707502 10.1007/s00520-019-05121-4 PMC12243493

[25] Xiaobin L Yan L. A Meta-ethnographic-based theoretical model of family caregiving experience at the end of life. *J Nurs Contin Educ.* (2023) 38:97–103.

[26] Alam S Hannon B Zimmermann C. Palliative care for family caregivers. *J Clin Oncol.* (2020) 38:926–36. 32023152 10.1200/JCO.19.00018

[27] Aapro M Caprariu Z Chilingirov P Chrápavá M Curca RO Gales L Assessing the impact of antiemetic guideline compliance on prevention of chemotherapy-induced nausea and vomiting: results of the nausea/emesis registry in oncology (NERO). *Eur J Cancer.* (2022) 166:126–33. 35290913 10.1016/j.ejca.2022.01.028

[28] Li Y Sun Y Liu B Sun Y Chen P Xie K Prolonged administration of aprepitant improves cisplatin-based chemotherapy-induced nausea and vomiting. *Future Oncol.* (2022) 18:2533–43. 35587019 10.2217/fon-2021-1523

[29] Wei H Sun Y Xie L Jia Y He J Deng X Huo Xiang Zheng Qi Oral liquid combined with 5-HT3 receptor antagonists and dexamethasone can prevent chemotherapy-induced nausea and vomiting for patients receiving multiday cisplatin-based regimen: a multicenter trial. *J Integr Complement Med.* (2023) 29:501–9. 36999940 10.1089/jicm.2022.0672

